# Development of Phosphorothioate DNA and DNA Thioaptamers

**DOI:** 10.3390/biomedicines5030041

**Published:** 2017-07-13

**Authors:** David E. Volk, Ganesh L. R. Lokesh

**Affiliations:** McGovern Medical School, Brown Foundation Institute of Molecular Medicine for the Prevention of Human Diseases, University of Texas Health Science Center, Houston, TX 77030, USA; lokesh.rao@uth.tmc.edu

**Keywords:** DNA aptamer, thioaptamer, X-aptamer

## Abstract

Nucleic acid aptamers are short RNA- or DNA-based affinity reagents typically selected from combinatorial libraries to bind to a specific target such as a protein, a small molecule, whole cells or even animals. Aptamers have utility in the development of diagnostic, imaging and therapeutic applications due to their size, physico-chemical nature and ease of synthesis and modification to suit the application. A variety of oligonucleotide modifications have been used to enhance the stability of aptamers from nuclease degradation in vivo. The non-bridging oxygen atoms of the phosphodiester backbones of RNA and DNA aptamers can be substituted with one or two sulfur atoms, resulting in thioaptamers with phosphorothioate or phosphorodithioate linkages, respectively. Such thioaptamers are known to have increased binding affinity towards their target, as well as enhanced resistance to nuclease degradation. In this review, we discuss the development of phosphorothioate chemistry and thioaptamers, with a brief review of selection methods.

## 1. Introduction

Nucleic acid aptamers are short segments of single-stranded DNA or RNA that mimic antibodies in their ability to specifically recognize and bind cognate target molecules with high affinities [1,2,3]. The aptamer field began in 1990 with the work of three groups. First, Tuerk and Gold [1] described an in vitro iterative process of RNA-T4 DNA polymerase binding experiments, with reverse transcription polymerase chain reaction (RT-PCR) amplification between each round, to progress from a starting RNA library with an eight-nucleotide random region down to two highly enriched RNA sequences. They named this process Systematic Evolution of Ligands through EXponential Enrichment (SELEX). Robertson and Joyce used a similar technique for the evolution of an RNA enzyme that cleaves ssDNA [2]. Ellington and Szostak [3] used a random RNA library containing 10^13^ sequences to develop the first aptamers targeting small molecules, specifically organic dyes. Many new aptamer selection and separation techniques have been developed since that time, and they have been reviewed in detail [4,5,6,7,8].

Aptamer selection methods have now been described using whole cells [7,9,10,11,12], excised tissues [13,14] and even live animals [15,16], because aptamers selected in vitro do not always work well in vivo [17,18]. Whereas typical SELEX is designed to produce aptamers targeting a known protein, the Cell-SELEX method targets the cells of interest directly, without the need for a predefined and purified target protein. It can be used to find aptamers that bind to the cell surface or become internalized. The first Cell-SELEX experiments targeted *Trypanosoma brucei* (*T. brucei*) [19,20] and *Trypanosoma cruzi* (*T. cruzi*) [21,22], although it has also been suggested [7] that earlier work targeting membranes from red blood cells [23] was the first Cell-SELEX experiment. The technique was subsequently used to derive aptamers targeting mammalian embryonic stem cells [24,25], infected cells [26] and cancerous cells [27]. Although Cell-SELEX has the advantage of not requiring a predefined target protein, it has numerous disadvantages. Cell-SELEX targets a cell surface decorated with a complex mixture of proteins and their post-translational variants, common to many cell types. Thus, it requires additional negative selection experiments against multiple common cell types. Secondly, lab-grown cells are a mixture of live and dead cells, so separation of these two cell types is warranted. Cell-SELEX is an excellent method for finding new biomarkers for specific cell types, and this is particularly true in cancer, as recently reviewed in 2016 by Chen et al. [7] and previously in 2014 by Sun et al. [28] and by Sun and Zu [29] in 2015. Live-animal-based-SELEX (also called in vivo-SELEX) has also been used to overcome the problems associated with in vitro protein-SELEX [17,18]. Mi et al. first used tumor-bearing mice as the selection medium to derive RNA aptamers targeting intrahepatic colorectal cancer and found an aptamer that targets p68, an RNA helicase [15]. Subsequently, Cheng et al. used this method to derive brain-penetrating aptamers [16], and just last year an aptamer targeting RNA helicase DHX9 [30]. Mai et al. used in vivo SELEX to derive DNA aptamers targeting lymphoma [31]. Tissue slide-based-SELEX is an intermediate method between selecting against live cells and live animals. In 2009, Li et al. first used the tissue slide-based-SELEX [13] method to develop an aptamer targeting hnRNP A1. Subsequently, Wang et al., in a method they call Morph-X-Select, incorporated the use of laser-microdissection to enable more precise morphology-based selection of aptamers or thioaptamers directly from pathological tissue slices [14]. They were able to simultaneously select aptamers (to unknown targets) and discover new biomarkers from tissue-bearing ovarian cancer, and do so for both the tumor vessels and the tumor cells.

A variety of techniques has been developed to improve the separation of aptamer:target complexes from unbound aptamers and target. Capillary electrophoresis separation methods (CE-SELEX [32,33,34,35] and nonequilibrium capillary electrophoresis of equilibrium mixtures (NECEEM) [36]) can remove the need for polymerase chain reaction (PCR) amplification and provide aptamers in as little as one round of selection. Likewise, other non-SELEX methods with separations based on chromatography (MonoLEX) [37,38], bead libraries [39], microfluidics (mSELEX) [40,41,42], atomic force microscopy (AFM) [43,44], graphene oxide (GO-SELEX) [45,46,47], surface plasmon resonance (SPR) [48,49,50] or fluorescence-activated cell sorting (FACS) [51,52] can reduce the time requirements and number of selection rounds required to obtain aptamers.

In order to create aptamers with enhanced stability to make them more relevant in the clinic, a variety of chemical modifications has been utilized to improve their in vivo life-times. Macugen (pegaptanib), a pegylated RNA aptamer that acts against vascular endothelial factor, was the first nucleic acid aptamer therapeutic approved by the United States Food and Drug Administration (FDA) for the treatment of age-related macular degeneration [53,54,55,56], and it contains 2′-fluoropyrimidines nucleotides and 2′-methoxy purines [56]. The use of 2′-fluoropyrimidines is fairly common [56,57,58]. Other 2′-ribose substitutions include 2′-amino, 2′-azido, 2′-hydroxymethyl and 2′-thio [59,60,61,62]. Other sugar modifications include bicyclic ribose derivatives in which the 2′- and 4′-positions are linked together forming blocked/locked nucleic acids [63,64,65], threose nucleic acids (TNA) [66,67], cyclohexenyl nucleic acids (CeNA) [68], glycerol nucleic acids (GNA) [69] and peptide nucleic acids (PNA) [70]. The phosphate backbones of aptamers have also been stabilized with the introduction of boranophosphates [71] and thio-/dithio-phosphates [72,73,74,75], which are the focus of this review.

## 2. Chemistry of Phosphorothioate and Phosphorodithioate DNA Backbones

**Figure 1:**
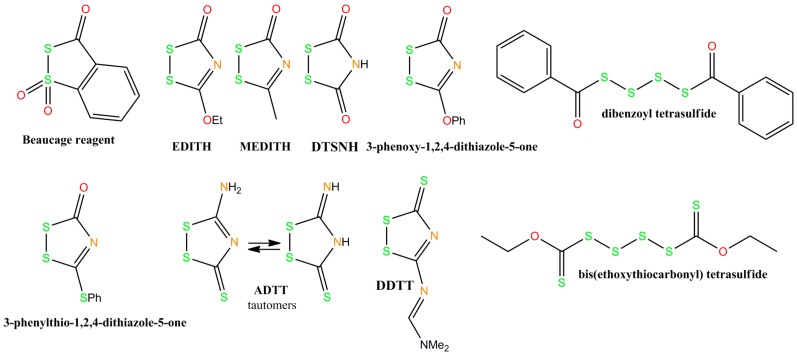
Chemical structures of selected oxidative sulfurization reagents. EDITH: 3-ethoxy-1,2,4-dithiazolidin-5-one; MEDITH: 3-methyl-1,2,4-dithiazolin-5-one; DTSNH: 1,2,4,-dithiazolidine-3,5-dione; ADTT: 3-amino-1,2,4-dithiazole-5-thione; DDTT: 3-((dimethylamino-methylidene)amino)-3H-1,2,4-dithiazole-3-thione.

**Scheme 1:**
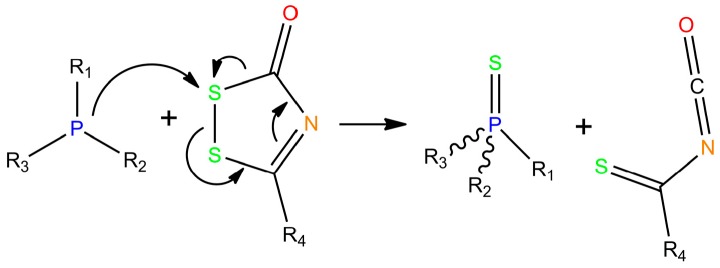
Oxidative sulfurization of a phosphotriester with a 1,2,4-dithiazole-5-one.

The first phosphorothioate dinucleotide linkage was reported by Eckstein in 1967 [76], and he subsequently showed that RNA with phosphorothioate linkages is more resistant to ribonucleases (RNAses) compared to regular RNA [77]. This finding led to increased interest in sulfurization of oligonucleotides. A variety of sulfurizing reagents (Figure 1) has been used to synthesize phosphorothioate linkages from phosphite triesters, including elemental sulfur [78], tetraethylthiuram disulfide (TETD) [79], 3H-1,2-benzodithiol-3-one 1,1-dioxide also called Beaucage reagent [80], dibenzoyl tetrasulfide [81], bis(*O*,*O*-diisopropoxy phosphinothioyl)disulfide [82], 3-ethoxy-1,2,4-dithiazolidin-5-one (EDITH) [83], 1,2,4,-dithiazolidine-3,5-dione (DTSNH) [83], 3-methyl-1,2,4-dithiazolin-5-one (MEDITH) [84], 3-amino-1,2,4-dithiazole-5-thione (ADTT) [85] and, more recently, 3-((dimethylamino-methylidene)amino)-3H-1,2,4-dithiazole-3-thione (DDTT) [86]. 3-phenoxy 1,2,4-dithiazole-5-one and 3-phenoxy-1,2,4-dithiazole-5-one are also highly reactive oxidative sulfurizing agents, similar to EDITH [87]. Many of them contained a common five-membered ring system with a disulfide bond that undergoes a ring-opening reaction that starts with nucleophilic attack of the sulfur atom near the carbonyl carbon (Scheme 1).

For a number of years, the Beaucage reagent [80] was widely used because it was inexpensive and stable when dry, but it suffered from stability problems once it was solvated and placed on a synthesizer. DTSNH and its precursor EDITH [83] gained popularity because they are very effective oxidative sulfurizing agents when using a short 2-min oxidation step, even at low (0.05 M) concentration. Ma et al. [88] found that when EDITH was used with fast deprotection methods (*t*-butylphenoxy acetyl protecting groups) and a capping step before the thio oxidation step, it provides oligonucleotides with fewer undesirable G substitutions and that mild deprotection can be used to synthesis oligonucleotides, such as thioated RNA, that are unstable to deprotection in concentrated ammonium hydroxide at elevated temperatures. An equally effective, but less expensive reagent, ADTT [85] was introduced in 2000 that offered similar benefits, but at a lower cost [85]. At present, DDTT [86] is often the preferred reagent for sulfurization due to its combination of low cost, reaction kinetics, efficacy, stability in a solvated form and its excellent oxidative sulfurization of RNA [89], but EDITH is also still used. DDTT can be shipped to the end user as a pre-made 0.1 M solution. As noted by Ponomarov et al., the 1,2,4,-dithiazole-5-ones are at least two orders of magnitude more reactive than the 1,2,4-dithiazoles [87]. Therapeutic phosphorothioates were recently reviewed by Eckstein [90]. At present, synthesizing oligonucleotides with phosphoromonothioate substitutions is easily performed on standard DNA and RNA synthesizers using solid-phase phosphoramidite chemistry [91], developed by Caruthers, and oxidative sulfurization in place of the standard oxidation step using iodine.

**Figure 2:**
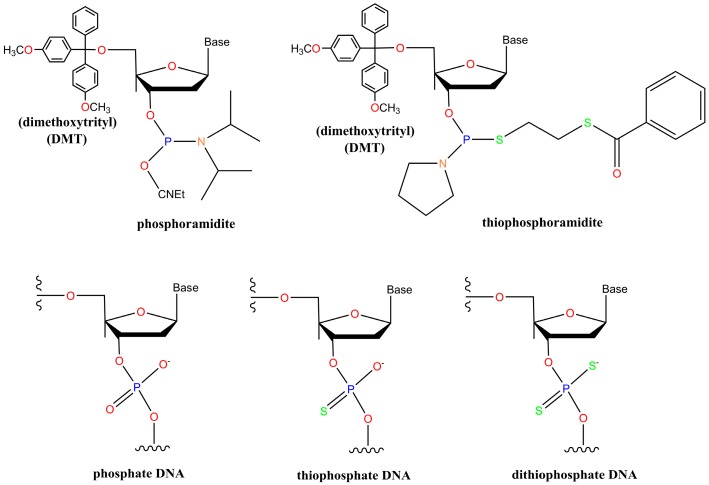
Structures of DNA synthesis reagents and DNA containing normal DNA backbones or thioated DNA backbones.

As phosphoromonothioate oligonucleotides became more common, the search for methods to create phosphorodithioate oligonucleotides began. In 1993, two groups led by Gorenstein [92] and Caruthers [93] were awarded patents for the production of oligonucleotides containing dithiophosphate substitutions using thiophosphoramidites (Figure 2) in place of phosphoramidites, and subsequent oxidative sulfurization. A handful of improvements in the method, mostly in regards to the phosphoramidite leaving group, were patented in the following few years by Caruthers’ group [94,95,96,97,98] and are described in multiple publications [99,100,101,102]. Solid-phase synthesis of dithioate oligonucleotides has recently been reviewed [103]. As the chemistry and understanding of both monothioate and dithioate DNA/RNA improved and the field of aptamer selection matured, the two fields began to merge.

## 3. Thioaptamer Development

### 3.1. The Beginning

David King et al. conducted the first combinatorial DNA thioaptamer selection experiments, using α-S-dATP in place of the usual dATP nucleotide during PCR, targeting the nuclear factor for interleukin 6 (NF-IL6), a basic leucine zipper involved in the inflammation cascade [104]. In this work using a 22-nucleotide random region flanked by two primers, they found that the thioaptamer out-competed the native binding sequence for NF-IL6 and bound in a two-to-one ratio, unlike the native binding 10-mer, which binds NF-IL6 at a one-to-one ratio.

Around this same time, Yang et al. investigated short decoy thioated DNA in the form of the monothioated and dithioated CK14 aptamer decoy binding to nuclear factor kappa B (NF-κB). In this work, it was shown that the binding characteristics of phosphorodithioates [105] were superior to both phosphoromonothioates and phosphate-only versions of CK14 and that structural changes occur in the DNA with the duplex taking on a more A-DNA-like structure [106]. Later ab initio calculations suggested that decreased binding of sodium atoms to the thioated phosphates may account for the increased binding properties of phosphorothioates and phosphorodithioates [107]. Marshall and Caruthers had also demonstrated previously that phosphorodithioate DNA oligomers strongly inhibit human immunodeficiency virus type-1 reverse transcriptase (HIV-1 RT), relative to non-thioated oligonucleotides [108]. This enhanced binding of dithioated DNA leads to such a strong binding interaction between heavily-dithioated DNA and hydrophobic columns typically used to purify them, that purification of the full-length oligonucleotide with trimethylamine acetate buffer at room temperature is often poor, even with the dimethoxytrityl group still attached. Separation of full-length oligonucleotides from shorter synthetic failures missing a few non-dithioated bases on the 5′-side is inadequate. To overcome this, Xin Bin Yang et al. devised a better purification scheme using anion-exchange chromatography with sodium thiocyanate salt [109]. The unusual binding strength of dithioates is exemplified by a report from last year, when Abeydeera et al. demonstrated that a single dithioate substitution in an α-thrombin RNA aptamer produced a 1000-fold improvement in binding [110]. As shown recently by Wu et al., the combination of dithioate DNA linkages and 2′-*O*-methyl substitutions enhanced the anti-tumor activity of a 25-base pair siRNA molecule by forming a more stable RISC complex [75].

Following their NF-IL6 thioaptamer selection experiments, King et al. used the same thioaptamer selection procedure to target the NF-κB proteins p65 (also called RelA)) and p50 [111]. With binding constants of 1–5 nM, the thioaptamers could effectively compete with the native binding element, immunoglobulin kappa B (IgκB), which has a weaker binding constant of ~82 nM, and the inflammation cascade could be altered by treatment with the NF-κB thioaptamer.

In exploring ways to improve the standard in vitro combinatorial aptamer selection process used at the time, which requires as many as 10–15 rounds of selection and PCR amplification, Yang et al. constructed the first bead-based thioaptamer/dithioaptamer selection process [112]. Using fluorescently-tagged proteins, the beads containing the best binding aptamers could be easily, albeit slowly, selected by hand under fluorescent lighting or they could be sorted in a high-speed manner using flow cytometry via fluorescence-assisted cell sorting (FACS) methods [39], as is also done with cell-based FACS-SELEX [51,52]. Although the bead sequences were read out by PCR of the sequences attached on the selected beads, an important feature is that amplification of the library was not required as part of the selection process.

### 3.2. Thioaptamers Targeting Infectious Diseases

In 2005, Anoma Somasunderam et al. developed double-stranded DNA thioaptamers targeting the ribonuclease H (RNase H) domain of human immunodeficiency virus-1 reverse transcriptase (HIV-RT) and demonstrated inhibition of the HIV-RT and anti-viral activity in vitro [113], in a dose-dependent manner. Although the top thioaptamer binds to the isolated RNase domain of HIV-RT, it did not bind to the RNase H domain from *Escherichia coli*, indicated that it is a sequence-specific aptamer. Subsequently, Ferguson et al. tested several delivery routes of the double-stranded aptamer and reported viral inhibition as high as 83% [114].

In 2006, Vaillant et al. tested the anti-inhibitory effects of three classes of random sequences (randomers) [115]. Randomer 1 sequences contained phosphorothioate DNA bases; Randomer 2 contained phosphorothioate, 2-*O*-methyl RNA bases; and Randomer 3 contained 2-*O*-methyl RNA bases only. They found that the phosphorothioate sequences bound to the heptad repeats on gp41, presumably on the hydrophobic face of this fusion peptide, leading to anti-viral activity. In contrast, the 2-*O*-methylated RNA did not lead to anti-viral activity, despite also binding to gp41, presumably on the hydrophilic face of the fusion peptides.

In 2007, Prusiner’s group investigated the effects of phosphorothioate DNA on the infectious prion protein PrP^Sc^ [116,117]. Karpuj et al. found that phosphorothioated 22-mers decreased levels of PrP^Sc^ and PrP^C^ in prion-infected (scrapie-infected, Sc) neuroblastoma (N2a) cells (ScN2a) regardless of the DNA sequence [116]. The thioated DNA EC_50_ for half-maximal decrease of PrP^Sc^ was 50-times lower than the EC_50_ for PrP^C^, illustrating preferred action against the disease-causing form of the prion protein. In another paper from Prusiner’s group, published in the same month, King et al. [117] describe a thioaptamer family selected against Syrian hamster (Sha) recombinant prion protein (recSHaPrP). This family of aptamers contained a consensus sequence of twelve bases containing five critical monothioated deoxyadenosine (dA) bases. Their best aptamer bound to recSHaPrP with a 0.6 nM Kd, but with less affinity to PrP from bovine or human sources, thus showing protein sequence specificity.

Encouraged by earlier work targeting *T. brucei* and *T. cruzi* [19,20,21,22], Fennewald et al. demonstrated the in vivo utility of thioaptamer decoys for controlling the effects of viral hemorrhagic fever viruses in the arenavirus group [118]. In particular, studying the Lassa virus and Pichinde Virus (PICV), they demonstrated that their decoy thioaptamer called XBY-S2 bound to Fos-related antigen 2 (Fra-2) and Jun B, thereby enhancing cytokines levels (interleukin-6 (IL-6), interleukin-8 (IL-8), tumor necrosis factor alpha (TNF-α)) and greatly reducing mortality in guinea pigs infected with legal doses of Pichinde virus. When treated prophylactically with the XBY-S2 thioaptamer and again on Day 2 post-infection, 61% of the animals survived the PICV infection while only 21% of the untreated animals survived. Treatment with thioaptamer XBY-S1, which contains the same number of dithioate bases as XBY-S2, provided no significant change in survival.

In a more recent related study, Liu et al. described 20-mer dsDNA thioaptamers targeting the AP-1 transcription factor protein [119]. The aptamers contained either 3, 4, or 5 phosphorothioate end cap (EC) substitutions at each end of the thioaptamers and fluorescein isothiocyanate (FITC), gold or superparamagnetic iron oxide nanoparticles (SPIONs) for detection by optical imaging, transmission electron microscopy (TEM) or magnetic resonance imaging (MRI). In this study using C57black6 mice and a mutant with an altered A-Fos protein with enhanced binding affinity to Jun, they demonstrated multimodal imaging of transcription factor trafficking in the brain.

Kang et al. developed an RNA thioaptamer that targeted the capsid protein of the Venezuelan equine encephalitis (VEE) virus with a binding constant of about 70 nM in 2007 [120]. More recently, Gandham et al. developed a thioaptamer targeting the purified envelope protein domain III (ED3) of the Dengue 2 virus [121], but it has failed to reduce virus titers when tested against live cells up to this time.

### 3.3. Cancer-Related Thioaptamers

In 2008, Kang et al. developed a thioaptamer targeting tumor growth factor β-1 (TGF-β1) [122]. Surprisingly, despite the use of both thio-dA and thio-dC in the thioaptamer selection process, thus creating a heavily-monothioated aptamer, the TGF-β1 thioaptamer’s binding constant was only 90 nM. In 2015, Mathura et al. incorporated this thioaptamer into an aptasensor, which could detect TGF-β1 over a linear range of 1–250 ng/mL [123]. As part of a microfluidics device, the sensor could monitor the TGF-β1 release from activated hepatic stellate cells for nearly a day. Zhou et al. [124] had shown a similar lower level of detection for thrombin using two aptamers, named aptamer 15 (Apt15) [125] and aptamer 29 (Apt29) [126], targeting different epitopes of thrombin.

Somasunderam et al. developed a thioaptamer targeting the CD44 hyaluronic acid binding domain, or CD44 HABD [127]. The CD44 protein plays a large role in tumor growth and metastasis and is the primary binding site of hyaluronic acid. Although CD44 has many splice variants, its HABD is invariant and thus served as an excellent target for thioaptamer binding. In this work, they developed thioaptamers, with binding constants in the 200–300 nM range, which were able to block the binding of hyaluronic acid because hyaluronic acid only binds to CD44 HABD with a micromolar binding constant. The thioaptamers exhibited excellent binding to three human ovarian cancer cell lines—SKOV3 (Sloan-Kettering ovarian cancer-3), IGROV-1 (Institut Gustave Roussy Ovarian cancer-1) and A2780 (ovarian adenocarcinoma), but did not bind to CD44-negative cells, suggesting that they could be useful targeting agents against cancer. In 2016, Fan et al. demonstrated safe delivery of siRNA using a CD44 thioaptamer in a dendritic polyamidoamine system in vivo [128].

Another cancer-related DNA thioaptamer, called ESTA1 (E-Selection ThioAptamer 1), was selected against the soluble portion of the E-selectin protein [129]. The NF-κB and AP-1-inducible glycoprotein E-selectin (also called CD62 antigen-like family member E (CD62E), endothelial leukocyte adhesion molecule-1 (ELAM-1) or leukocyte-endothelial cell adhesion molecule-2 (LECAM-2)) is upregulated in the inflamed vasculature associated with many cancers, including, ovarian, breast and colon cancers, while not being constitutively expressed in endothelial cells [130]. The selectin proteins, including E-, L- and P-selectin, are surface glycoproteins that play a role in leukocyte tethering and rolling along endothelial walls [131,132]. Thus, they are attractive targets for localized delivery of cytotoxic chemotherapy drugs.

In 2010, Mann et al. showed that ESTA1 could be used to guide biodegradable mesoporous silicon microparticles to the bone marrow vasculature as part of a multifunctional drug delivery system for imaging or for therapeutic purposes [133]. In 2011, Mann et al. also showed that liposomes conjugated to ESTA1 could target inflamed vasculature associated with cancer [134]. Zong et al. demonstrated targeted delivery of ESTA-1-labeled multistage drug delivery vehicles to acute myelogenous leukemia (AML) blasts and leukemia stem cells [135] and reduced AML tumor burden by up to 60% while greatly reducing drug dosage and dosing frequency [136]. Later, Mai et al. demonstrated the therapeutic effects of the ESTA1-conjugated multistage vector (ESTA-MSV) drug carrier for the treatment of metastatic breast cancer in bones by targeting the bone marrow [31]. This directed delivery of siRNA targeting the *STAT3* gene (ESTA-MSV/*STAT3* siRNA) knocked down STAT3 protein production in nearly 50% of cancer cells in the bone and led to enhanced survival (25% extension of life) in mice bearing MDA-MB-231 bone metastasis. It was then shown that ESTA1 can be used to block the metastatic-niche and reduce the spread of breast cancer metastasis in the lungs [137] and that it does so in a safe manner [138]. This work implies that ESTA1 may work in a variety of other hematogenous metastasis. It was also shown that PEGylation of ESTA1 provides enhanced circulation time for this aptamer [139].

An RNA aptamer [140] stabilized with 2′-amino pyrimidine nucleotides and a DNA aptamer [141] targeting L-selectin have been reported. Although the RNA and DNA aptamers targeting L-selectin were both effective at blocking the interaction between L-selectin and sialyl Lewis X carbohydrate, only the DNA aptamer was also capable of blocking lymphocyte trafficking in vivo in severe combined immune deficiency (SCID) mice containing the *scid* mutation. Although the RNA aptamer affinity to L-selectin was high at lower temperatures, its weak interaction at 37 °C made it unsuitable for in vivo testing.

Gutsaeva et al. produced an anti-mouse P-selectin RNA aptamer (ARC5690) stabilized with 2′-fluoro pyrimidines and 2′-methoxy purines [142] and tested it both in vitro and in a sickle cell disease (SCD) transgenic mouse model. In vitro tests indicate that the core of the aptamer binds with an impressive Kd of ~15 pM with little to no binding with E-selectin. In SCD mice dosed at 20 mg/kg of body weight via intraperitoneal injection, the ARC5690 aptamer outperformed an anti-P-selectin antibody in anti-adhesion tests and performed comparably to the antibody in red blood cell (RBC) velocity measurements and the reduction of leukocyte rolling flux. In a hypoxia/normoxia stress experiment, SCD mice treated with aptamer ARC5690 had a higher survival rate (86%, 12/14) compared to saline-treated (67%, 14/21) or scrambled aptamer-treated mice (53%, 8/15).

In 2012, Watanabe et al. tested the effects of DNA thioaptamers targeting high mobility group A (HMGA) proteins HMGA1 and HMGA2 in pancreatic cancer cell lines [143]. These proteins are architectural transcription factors known to bind in the minor groove of adenosine:thymine (A:T) tracks [144], and high expression levels of HMGA1 are associated with chemotherapy resistance [145]; while HMGA2 is associated with Ras-induced invasion and metastasis, and its downregulation inhibits pancreatic cell lines [146]. Most notably, the AT-rich phosphorothioate aptamer (AT-sDNA) increased the sensitivity of Miapaca-2 and AsPC-1 pancreatic cancer cell lines to gemcitabine treatment, presumably by acting as a decoy to bind to excess HMGA1 [143].

In 2015, Hu et al. described selecting a thioaptamer containing thio-dA, named HY6 [147], against an extracellular domain (ECD) peptide of the human epithelial growth factor receptor 2 (HER2, ErbB2). HER2 overexpression is associated with many cancers, including breast cancer [148], lung cancer [149], ovarian cancer [150] and gastric and esophageal carcinomas [151], and treatment targeting HER2 with antibodies is known [152,153]. HY6 binds to the extracellular domain of HER2 with a binding constant of 178 nM, and they showed binding to HER2-positive SK-BR-3 and MDA-MD-453 breast cancer cells, but not to HER2-negative MDA-MB-231 cells. Liu et al. had previously developed a non-thioated aptamer (HB5) targeting a HER2 ECD peptide [154]. HB5 was found to bind the ECD of HER2 with a 316 nM Kd and bind more tightly to an epitope peptide with an 18.9 nM Kd [154]. In vitro testing of a HB5-doxorubicin complex showed preferential treatment to HER2-positive breast cancer cells. The HB5 and HY6 sequences have some similarity in that they both contain T stretches. While the thioaptamer HY6 was stable in serum and was able to bind its target after long exposure in serum, its analog without thiophosphate substitutions could not [147].

In 2016, Mangala et al. described the selection of thioaptamers targeting ovarian cancer-associated endothelial cells generated by using a thioaptamer version of the Cell-SELEX technique [155] against ovarian cancer associated-endothelial cells derived from ten different patients while using cells derived from normal ovaries as controls. They found that the Endo28 thioaptamer targets annexin A2, a protein upregulated in cancer [156,157]. When Endo28 was conjugated to the surface of Chitosan (CH) Nanoparticles (NP) containing siRNA targeting micro-RNAs miR106b-5p and miR30c-5p, the CH/Endo28-siRNA-NP provided enhanced targeting of the tumor relative to other organs. Most striking was the near elimination of particle accumulation in the liver, which absorbs the majority of untargeted particles. Targeted delivery of these particles into orthotopic ovarian cancer models led to enhanced maturation of the vasculature surrounding the tumor and led to anti-tumor effects, suggesting that this therapy could lead to better therapy targeting of angiogenesis. The Endo28 thioaptamer has been connected to one arm of a stable three-Way Junction (3WJ) RNA from phi29 packaging RNA to form stable DNA/RNA hybrid nanoparticles that could be loaded with doxorubicin for targeted delivery to ovarian cancer cells [158]. The Endo28-3WJ-Sph1/Dox particles were delivered to annexin-2-positive IGROV-1 (71%) and SKOV-3 (52%) ovarian cancer cell lines, with very little binding (17%) to annexin-A2-negative human embryonic kidney 293 (HEK293) cells. In vivo testing showed that 6 h post-injection of 10 µM particles, the fluorescently-labeled particles were only detected in the xenograft tumors, demonstrating their potential utility as a targeting agent for annexin-2-positive cancers [158].

In 2015, Wu et al. developed two DNA aptamers, XQ-2 and a truncated version called XQ-2d, targeting Pancreatic Ductal Adenocarcinoma (PDAC) [159], the most common pancreatic adenocarcinoma [160]. The longer original aptamer, XQ-2, was selected against the PDAC PL45 cell line using Cell-SELEX, and it was shown to bind tumor sites in athymic BALB/c nude mice. They created both phosphorothioate and 2′-OMe derivatives of XQ-2d to increase serum stability, but found that the thioaptamer version of XQ-2d exhibited reduced binding to PL45 cells, while the 2-OMe version did not.

AXL-tyrosine receptor kinase plays a role in many cancers, including breast [161], lung [162], ovarian [163], pancreatic [164] and prostate [165] cancers, as well as leukemia [166]. In 2012, Cerchia et al. developed GL21.T, an RNA aptamer targeting the AXL-tyrosine receptor kinase (AXL) protein [167]. They showed that GL21.T inhibits the formation of lung tumors in xenograft mouse models, presumably by binding to the extracellular domain of AXL and blocking downstream phosphorylation of Erk and Akt proteins. Because AXL downregulation has been shown to be an attractive therapeutic target for ovarian cancer [156,168,169,170,171], Kanlikilicer et al. created and tested a serum-stable DNA version of GL21.T, called AXL-DNA-APTAMER, containing both 2′-fluoropyrimidine nucleosides and thiophosphates [172], and tested its effects on ovarian cancer. In two models of intraperitoneal ovarian cancer, the AXL-DNA-APTAMER suppresses metastasis, migration/invasion and tumor growth on its own and enhances the effects of paclitaxel treatment [172], presumably by disrupting focal adhesion kinase (FAK) phosphorylation and matrix-metalloproteinases. These aptamers may serve as alternate future treatments for ovarian cancers, especially those that have become drug-resistant.

### 3.4. Other Thioaptamers

In 2012, Lui et al. described an aptamer they called Adipo8 that specifically targets mature white adipocytes with an 18 nM Kd [173]. Using the Cell-SELEX procedure, they first targeted differentiated 3T3-L1 adipocytes before doing counter selection with undifferentiated 3T3-L1 preadipocytes or HepG2 cells during aptamer selection rounds. They found that Adipo8 did not bind to six types of cancer cells, B cells or T cells, nor to hepatocytes or muscle cells known to display some of the same proteins displayed on adipocytes after differentiation. More recently [174], Chen et al. tested a 5′-PEG-modified version of Adipo8 with phosphorothio linkages located just after the first base and just before the last base. The addition of these two thioate linkages increased the serum stability by a small, yet statistically significant amount when tested on tissue culture and in vivo. Because this aptamer can selectively distinguish white adipocytes from brown adipocytes and pre-adipocytes, it may serve as a useful delivery agent for adipocyte-specific therapy.

In 2013, Higashimoto et al. derived the first thioaptamers, containing both thio-dA and thio-dT, targeting Advanced Glycation End-products (AGEs) [175], and found that three of them, #4s, #7s and #9s, bound to AGE-Human Serum Albumin (HSA) with subnanomolar binding. Elevated levels of AGEs and interaction with their Receptor (RAGE) increase vascular hyperpermeability and thrombosis by inducing Vasculature Endothelial Growth Factor (VEGF) and Plasminogen Activator Inhibitor-1 (PAI-1) [175,176]. AGEs are associated with complications of diabetes, such as retinopathy, and other vascular and degenerative diseases, and suppression of AGEs is a potential therapeutic strategy [176]. Higashimoto et al. found that when Human Umbilical Vein Endothelial Cells (HUVECs) were exposed to 20 mM thioaptamer #4s, induction of RAGE, VEGF and PAI-1 was significantly reduced and that the aptamer inhibited binding to AGE-HSA in a dose-dependent manner [175]. In subsequent work [177], Maeda et al. tested the effects of an AGE-aptamer in a Streptozotocin (STZ)-induced diabetic Wistar rats using continuous intraperitoneal infusion and found that it prevented elevation in AGEs and abnormalities in Electroretinograms (ERGs). In earlier work, Higashimoto et al. [178] showed that a group of non-thioated aptamers targeting AGEs could inhibit apoptotic cell death and decrease DNA synthesis in pericytes and that one of them, clone 9, blocked the toxic effects of AGEs despite its relatively weak binding interaction (1 µM).

Mai et al. recently develop thioaptamers using in vivo-SELEX [31] to develop robust targeting agents for lymphoma. In this work, they demonstrated that metastases in the bone of living animals could be selectively targeted for treatment. Han et al. have also shown that thioaptamers attached to PEGylated Single-Walled Nanotubes (SWNTs) also provide targeting to breast cancer cells in vivo and in vitro [179].

## 4. Recent Developments in Thioaptamer Methods

### 4.1. Conjugate-SELEX

Most thioaptamer particle targeting experiments start by conjugating a known thioaptamer to a particle. However, recently, Mu et al. developed what they call the conjugate-SELEX method [180]. In this method, liposomes were created under dilute aptamer library conditions so that each liposome contained only one, or possibly a few, random DNA strands exposed on the surface. In each selection round, they collected the cytosolic fluid from head and neck squamous cell carcinomas. This method ensured that they were selecting only those thioaptamers that were successfully delivered to the cancer while attached to the liposomes.

### 4.2. X-Aptamers

**Figure 3:**
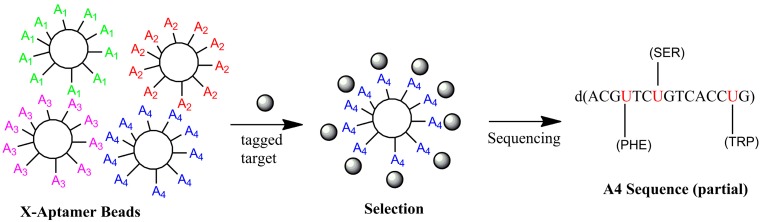
X-aptamer selection process using tagged proteins. Each aptamer bead contains many copies of a single X-aptamer. Beads are selected by fluorescence or magnets, and a second optional solution-phase selection step is not shown.

**Figure 4:**
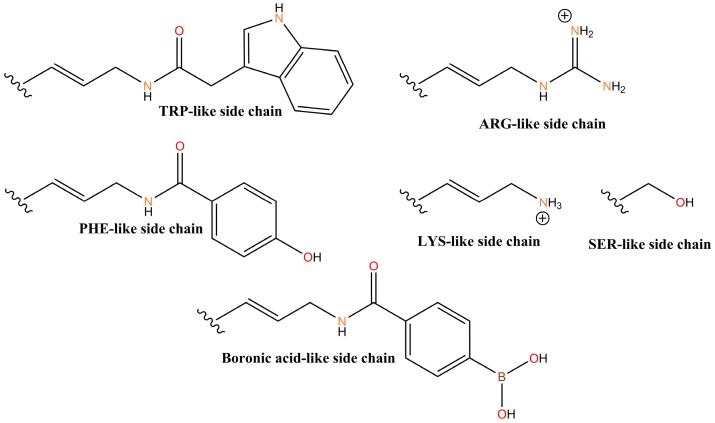
Example side chains (X) in 5-X-dU containing X-aptamer beads.

In 2012, He et al. [181] described X-aptamers (Figure 1) in which a DNA CD44 thioaptamer and a drug, which both bind to CD44 separately, were conjugated to create an improved binding agent. Combining the results of high-throughput computational drug screening, NMR binding verification, and a bead-based thioaptamer method [38] with a CD44 thioaptamer [121], a better binding reagent was created that was superior to either the drug or the thioaptamer alone. Specifically, a bead-based library of sequences with randomly-placed allylamino groups was created based on a CD44 thioaptamer [121], and these allylamino groups were conjugated to drug hits. Although other high-throughput analysis techniques, such as that reported by Thiel et al. [182], work well for random DNA/RNA libraries, Lu et al. developed Aptaligner [183] for the automated alignment and decoding of the pseudo-random X-Aptamer sequences. At present, over a dozen modified deoxyuridine reagents are available for use in aptamer synthesis and their relative merits have been reviewed recently [184]. A bead-based X-Aptamer Selection Kit (Figure 3), which contains a subset of these deoxyuracil derivatives (Figure 4) with protein-like side chains and others, is now available from AM Biotechnologies, LLC (Houston, TX, USA), and a detailed step-by-step protocol for its use will be available soon [185]. Despite misconceptions, the X-Aptamer process does not require the “SELEX” process, only DNA-protein binding and sequencing. The advantages include selection in one or two rounds and a diverse set of bases or drugs, but its major disadvantages are the pseudo-random nature of the designs and limited sequence diversity space (~10^8^ sequences) relative to traditional random libraries. Although the X-Aptamer method has similarities to slow off-rate modified aptamers (SOMAmers) [186], which were developed by Larry Gold’s group and which also contain protein-like side chains, X-Aptamers allow more freedom in that all occurrences of a particular base (dT/dU) are not required to all be modified, or all not be unmodified.

## 5. Conclusions

A wide variety of oligonucleotide substitutions are now available, and these methods have been exhaustively reviewed in several recent articles [7,8,184,187] and older publications [188,189,190]. The review by Zhou and Rossi published a few months ago is particularly thorough. While thioaptamers are becoming more prevalent, they still lag behind the development of RNA aptamers. Preclinical testing of these thioaptamers in animals shows the promise of future therapies in a number of diseases.
